# Comparison of phase-resolved functional lung (PREFUL) MRI and CT parametric response mapping (PRM) in COSYCONET COPD

**DOI:** 10.1007/s00330-026-12429-3

**Published:** 2026-03-28

**Authors:** Andreas Voskrebenzev, Marcel Gutberlet, Filip Klimeš, Lea Behrendt, Hoen-oh Shin, Hans-Ulrich Kauczor, Frank Wacker, Jens Vogel-Claussen, Till F. Kaireit

**Affiliations:** 1 Department of Diagnostic and Interventional Radiology, Hannover Medical School, Hannover, Germany; 2 Biomedical Research in Endstage and Obstructive Lung Disease (BREATH), Member of the German Lung Research Center (DZL), Hannover, Germany; 3 Department of Diagnostic and Interventional Radiology, Charité Universitätsmedizin Berlin, corporate member of Freie Universität Berlin and Humboldt-Universität zu Berlin, Berlin, Germany; 4 Department of Diagnostic and Interventional Radiology, University Hospital of Heidelberg, Heidelberg, Germany; 5 Translational Lung Research Center Heidelberg (TLRC), Member of the German Lung Research Center (DZL), Heidelberg, Germany

**Keywords:** Lung, COPD, Phase-resolved functional lung MRI, CT parametric response mapping, COSYCONET

## Abstract

**Objectives:**

To compare regional ventilation assessed by non-contrast enhanced ventilation-weighted phase-resolved functional lung (PREFUL) MRI with parametric response mapping (PRM) and with pulmonary function test (PFT) parameters in patients with chronic obstructive pulmonary disease (COPD).

**Materials and methods:**

This study was a retrospective analysis of a single-center subset of the prospective COPD cohort COSYCONET. PREFUL MRI coronal sections were obtained during free breathing at 1.5 T using a spoiled gradient echo sequence. PRM was derived from paired low-dose inspiratory and expiratory CT scans. Matched coronal slices of PREFUL and PRM were co-registered. PREFUL ventilation defect percentage (PREFUL-VDP), as well as functional small airway disease (PRM^fSAD^), emphysema (PRM^emph^), and their combined metric (PRM^fSAD+emph^), were calculated.

Global comparisons employed Spearman’s correlation coefficient (*r*) and Wilcoxon signed-rank tests. Spatial agreement was assessed using spatial overlap and the Dice coefficient.

**Results:**

Fifty-one patients (median age 65 [58–70]) were included in this study. PREFUL-VDP strongly correlated with combined PRM^fSAD+emph^ (*r* = 0.86. *p* < 0.001) and with PFT parameters (PREFUL-VDP vs FEV1, *r* = −0.75, *p* < 0.001). Correlations between PREFUL-VDP with PRM^fSAD^ and PRM^emph^ separately were weaker (*r* = 0.57 and *r* = 0.82, *p* < 0.001 for both). In concordance, the highest spatial congruence was observed between PREFUL-VDP and PRM^fSAD+emph^ (spatial overlap: 0.60 [0.55–0.66], Dice coefficient for defects: 0.53 [0.28–0.62]), indicating that PREFUL-VDP does not distinguish between small airway disease and emphysema.

**Conclusion:**

PREFUL-VDP correlates most strongly with the PRM measurement of emphysema and functional small airways disease combined and is a promising noninvasive, radiation-free tool for quantifying regional ventilation in COPD.

**Key Points:**

***Question*** *How does regional ventilation in COPD, measured by PREFUL MRI, correspond to CT-based PRM metrics?*

***Findings****PREFUL MRI’s VDP showed strong correlation and the highest spatial agreement with the combined CT PRM metric representing emphysema and functional small airways disease*.

***Clinical relevance****PREFUL MRI offers a non-invasive, radiation-free method for assessing regional ventilation in COPD. Although PREFUL cannot distinguish emphysema from small airways disease, its strong correlation with CT PRM highlights its value for disease characterization and functional monitoring*.

**Graphical Abstract:**

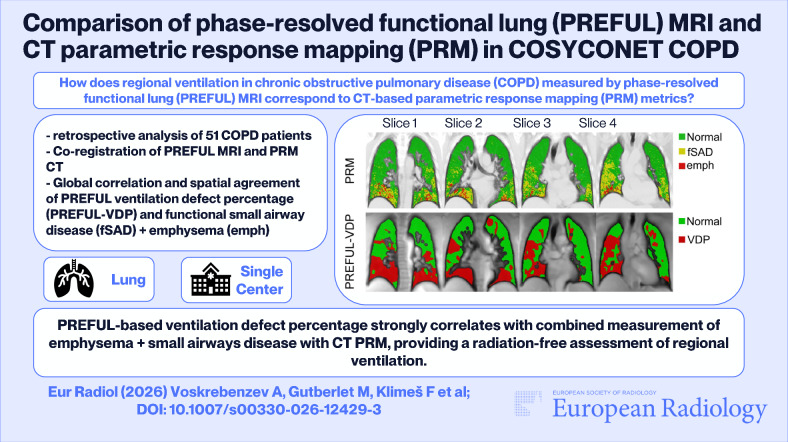

## Introduction

In recent years, efforts have been made to improve the phenotyping of chronic obstructive pulmonary disease (COPD) using lung imaging. Imaging biomarkers provide regional information beyond conventional lung function testing and have been linked with clinically relevant outcomes [1–10]. These biomarkers capture structural and functional changes of the airways and vasculature, which are hallmarks of COPD. However, due to the complex pathophysiological heterogeneity of the disease [2, 7], progress in defining and longitudinally monitoring the different components of COPD has remained limited [11].

Parametric response mapping (PRM) is a CT-based technique that uses radiodensity measurements of the lung to quantify the amount of normal (PRM^normal^), emphysematous lung parenchyma (PRM^emph^), and regions of air trapping due to functional small airways disease (PRM^fSAD^) [12]. This is achieved by co-registering inspiratory and expiratory CT scans and classifying each voxel according to two density thresholds. PRM has been shown to be reproducible over short time periods [11], to demonstrate spatial agreement with pulmonary gas MRI [13], to correlate with pulmonary function, and to associate with longitudinal decline in the forced expiratory volume in 1 s (FEV_1_) [14].

A limitation of PRM and other CT-based techniques is the requirement for ionizing radiation, which restricts their use for repeated examinations. This motivates the development of MRI-based methods for assessing regional lung ventilation.

Fourier Decomposition (FD) MRI, a proton-based postprocessing technique for dynamic free-breathing non-contrast enhanced lung MR imaging, allows assessment of lung perfusion and ventilation [15]. The basic principle of this approach is a frequency decomposition of voxel-wise signal time series, which separates proton density changes arising from lung parenchyma deformation during respiration from those related to pulmonary blood flow [16]. Phase-resolved functional lung (PREFUL) MRI extends this concept by retrospectively reordering images to reconstruct complete ventilation and perfusion cycles, thereby increasing the effective temporal resolution [17]. Previously, repeatability and reproducibility of ventilation-weighted FD and PREFUL MRI has been shown [18–20] and it has been validated against other techniques like single-photon emission computed tomography/CT [21, 22], Helium-3 (^3^He) MRI [23–25], ^129^Xe [26, 27], as well as ^19^F-MRI [28] and lung function testing in COPD patients [28]. More recently, PREFUL MRI has also been compared with PRM in asthma patients, showing a relationship between ventilation defects and PRM^fSAD^, but with little to no emphysema detectable by PRM in this cohort [29].

In contrast, a comparison between PREFUL MRI and PRM in COPD—where emphysema is a central pathological feature and PRM^emph^ contributes substantially—has not yet been reported. Therefore, in the present study, we investigate whether hypoventilation depicted by PREFUL MRI correlates and aligns regionally with PRM measurements, including emphysema burden, as well as with lung function test parameters in COPD patients.

## Materials and methods

### Patient characteristics and study protocol

The local ethics committee approved the study protocol. Written Informed consent was obtained from all participants after the nature of the procedure(s) had been fully explained.

Fifty-three patients with COPD were considered for inclusion in this retrospective analysis. All patients were originally enrolled in COSYCONET (“COPD and SYstemic consequenzes–COmorbidities NETwork”), a prospective, observational, multicenter cohort study in patients with stable COPD [5]. The present work represents a single-center subgroup analysis of COSYCONET, focusing on patients who underwent additional MRI examinations at our site.

Inclusion criteria were a COPD diagnosis by a pulmonologist according to the Global Initiative for Chronic Obstructive Lung Disease (GOLD) criteria [30] and an age of > 40 years. Exclusion criteria were previous chest surgery, pneumonia or moderate to severe exacerbations within the last 4 weeks, and contraindications to MRI or MRI contrast agents.

### Regional ventilation PREFUL MRI

PREFUL MRI was acquired as a single-center protocol amendment to the COSYCONET MRI protocol, which consists of morphological and contrast-enhanced sequences. All scans were acquired on a 1.5-T scanner (MAGNETOM Avanto, Siemens Healthineers).

For PREFUL four coronal sections, one at the middle of the tracheal level, two anterior and one posterior to the trachea, were acquired using an ungated spoiled gradient echo sequence with settings as follows: field of view 500 × 500 mm^2^, matrix size 128 × 96 (interpolated to 256 × 256), section thickness 15 mm, 7.5 mm gap between the section positions, echo time/repetition time 0.82 ms/3 ms, flip angle 5°, bandwidth 1500 Hz/px. Over a period of ∼1 min at a temporal resolution of 288 ms, 200 images per section were obtained. Total scan time for PREFUL MRI was ~4 min. The acquisition was carried out during free breathing and without a contrast agent.

Post-processing for the calculation of lung ventilation was performed as described by Voskrebenzev et al [17]. Briefly, all images were non-rigidly registered with the advanced normalization toolkit (ANTs) [31] towards one fixed image in intermediate lung position using the group-oriented registration scheme [32]. After lowpass filtering [32], temporal resolution was approximately increased to 33 ms (nominal) by sorting images according to their temporal phase of the respiratory cycle and subsequent interpolation to a uniform time grid. Finally, ventilation was quantified according to the fractional ventilation (FV) equation in terms of MR signal as introduced by Zapke et al:

$${FV}=\frac{{S}_{{Exp}}-{S}_{{Insp}}}{{S}_{{Exp}}},$$

with the MR signal in end-expiration and end-inspiration *S*_Exp_ and *S*_Insp_ during the synthesized ventilation cycle [33].

Afterwards, excluding the great central vessels, lobes of both lungs were segmented manually by a radiology resident (T.F.K.) with 4 years of experience at the time of segmentation. The middle lobe and lingula were counted to their corresponding upper lobes.

### PRM

After a brief breathing coaching, computed tomography of the lungs in full in- and expiration (i.e., total lung capacity (TLC) and residual volume (RV)) was performed in the supine position on a 64-section scanner (Lightspeed VCT, GE HealthCare) without i.v. contrast media on the same day as the MRI. Scan parameters for both respiratory states were as follows: tube current 120 kV, automatic tube modulation, table feed 39.375 mm/gantry rotation, 0.625 mm section thickness, and 0.7 mm reconstruction interval using a standard reconstruction kernel.

Lobes of both lungs were segmented semiautomatically by applying a local, adaptive region growing algorithm in inspiration and expiration using dedicated software (MeVisPULMO 3D, Fraunhofer MEVIS). The middle lobe and lingula were counted to their corresponding upper lobes.

The inspiratory datasets were registered onto the expiratory datasets using the non-rigid ANTs registration. PRM was carried out as described by Galban et al, labeling lung voxels according to two thresholds in expiration and inspiration as: PRM^normal^ (> −856 HU in expiration and > −950 HU in inspiration), PRM^fSAD^ (< −856 HU in expiration but > −950 HU in inspiration), and PRM^emph^ (< −856 HU in expiration and < −950 HU in inspiration) [12].

### Section-matching of PREFUL-MRI and PRM

Because the in-plane voxel size and section thickness of PREFUL-MRI were larger than those of the CT datasets, and both modalities were acquired with different respiration commands, dedicated steps were required for accurate section matching and analysis. Initially, the resolution of the CT data was downsampled by a factor of two in each dimension. Then, the center of the trachea, 10 mm cranial to the tracheal bifurcation, was semiautomatically identified in morphological CT and MRI images. The dorsoventral positions of CT and MRI sections were then calculated relative to this landmark, and the CT sections falling within each PREFUL-MRI section were identified, averaged, and used as reference images for inter-modal registration with ANTs (PREFUL MRI to CT).

For functional analysis, PRM maps were generated from the original (unaveraged) CT sections corresponding to each PREFUL section. These were combined into a PREFUL-matched PRM map by majority label voting applied in the *z*-direction, ensuring that each PREFUL section had a spatially aligned PRM counterpart. A consensual segmentation mask was created by merging the MRI- and CT-based masks, and this mask was applied to both PREFUL and PRM maps.

Thus, for each dataset, spatially aligned all-section and lobar median and interquartile range values were extracted. From PREFUL, the following metrics were obtained: FV (PREFUL-FV), the corresponding quartile coefficient of dispersion (PREFUL-QCD) as a measure of ventilation heterogeneity, and the relative volume of voxels classified as a ventilation defect (PREFUL-VDP). From PRM, percentages of PRM^normal^, PRM^fSAD^, and PRM^emph^ were derived, and a combined PRM-VDP was defined as the sum of PRM^fSAD^ and PRM^emph^ (equivalently, 1 – PRM^normal^).

Because lung ventilation in the supine position follows a physiological anterior–posterior gradient, ventilation defect maps for PREFUL were calculated using a section- and subject-specific threshold. For each section, the 75th percentile of parenchymal voxel values (representing normal ventilation) was determined and multiplied by 0.7, an empirical factor derived visually from five randomly selected COPD test cases spanning different GOLD stages. Voxels below this threshold were classified as ventilation defects. Similar distribution-based thresholding approaches have been applied previously [17, 34, 35].

Finally, combined CT-MRI match–mismatch maps were generated. These maps comprised four categories: consensus healthy (both modalities normal), PREFUL-only defect, PRM-only defect, and consensus defect (both modalities abnormal). For each category, median values of the difference in Hounsfield units between expiration and inspiration (ΔHU), defined as the difference between expiration HU and inspiration HU, as well as PREFUL-FV and PREFUL-QCD, were extracted to enable comparison between modalities in the respective regions.

### Validation of down-sampling and section matching

To ensure that the previously described matching steps did not introduce systematic bias, PRM values were compared under two conditions. First, to test the effect of spatial down-sampling, PRM metrics derived from full-resolution datasets were compared with those from down-sampled datasets in a subset of nine patients. Second, to assess the impact of restricting the lung volume to the PREFUL MRI section positions and applying majority label voting, PRM values from the full-lung CT datasets were compared with those obtained from the corresponding MRI-matched sections in all patients.

### Spirometry

Spirometry measurements were performed according to the American Thoracic Society and European Respiratory Society recommendations [36]. Data of the nearest visit in the study ambulance prior/after MRI (median 24 days; interquartile range 7–52 days prior to the MRI visit) was used for this study.

### Statistical analysis

The distribution of all functional MRI and CT parameters was tested for normality using the Shapiro–Wilk test. Since these variables were not normally distributed, nonparametric tests were used. Data are presented as median with 25th and 75th percentiles.

For cohort characterization, differences across GOLD stages were assessed using Kruskal–Wallis tests (continuous variables) and Fisher’s exact or exact Kruskal–Wallis tests (categorical or ordered categorical variables).

To evaluate potential bias introduced by PRM resampling, Bland–Altman analysis was performed, reporting mean differences and 95% confidence intervals (CIs).

Associations between PREFUL parameters, PRM metrics, and spirometry were analyzed using Spearman’s rank correlation on both global and lobar levels. For lobar-level correlations (nine tests in total), the significance level was adjusted to 0.006 using the Bonferroni method.

Comparisons across GOLD stages for PREFUL and PRM parameters were performed using the Kruskal–Wallis omnibus test, followed by Dunn’s test for post hoc analysis. Linear regression was applied to further assess agreement between PREFUL and PRM VDP values.

Agreement of VDP maps between PREFUL and PRM was evaluated by calculating spatial overlap and Dice coefficients for both defect and healthy voxel classes. In addition, the median values of the voxel classes were compared using the Wilcoxon signed-rank test.

Except where a Bonferroni adjustment was applied, a *p* < 0.05 was considered statistically significant. All analyses were conducted with JMP Pro 13 software (SAS Institute).

## Results

### Participant characteristics

Two of 53 patients were excluded from this study because of aborted MRI due to claustrophobia. Table 1 shows final demographic characteristics, GOLD distribution, and pulmonary function measurements for 51 patients with COPD (median age 65 (58–70) years), who successfully completed all examinations. Most demographic, spirometric, and imaging variables differed significantly across GOLD groups, whereas age, pack-years, and the number of exacerbations, antibiotic courses, and prednisolone courses did not.

**Table 1:** Subject demographics

Characteristic	All	GOLD I	GOLD II	GOLD III	GOLD IV	*p*-values
	(*n* = 51)	(*n* = 13)	(*n* = 17)	(*n* = 14)	(*n* = 7)	
No. of male subjects	23	4	7	7	5	**0.0051**^b^
Age (y)	65 (58–70)	67 (63–71)	64 (56–72)	63 (52–66)	66 (59–73)	0.1193^a^
Body mass index (kg/m^2^)	26 (23–30)	28 (23–32)	27 (25–31)	26 (21–29)	21 (19–25)	**0.0290**^a^
Packyears (y)	32 (17–50)	39 (29–52)	30 (5–51)	25 (16–33)	47 (34–92)	0.1027^a^
No. of former smokers	37	9	10	12	6	**< 0.0001**^b^
No. of current smokers	6	0	4	2	0	Tested together
No. of never smokers	8	4	3	0	1	Tested together
No. of A1AD	8	2	3	3	0	**0.0303**^b^
No. of exacerbations	1 (0–2)	0 (0–2)	1 (0–1)	1 (0–2)	1 (1–3)	0.6218^c^
No. of antibiotic courses	0 (0–1)	0 (0–1)	0 (0–1)	0 (0–0)	0 (0–1)	0.4332^c^
No. of prednisolone courses	0 (0–1)	0 (0–1)	0 (0–1)	0 (0–1)	1 (0–1)	0.4453^c^
FEV1 (% predicted)	52 (40–80)	89 (84–93)	56 (52–68)	42 (35–46)	23 (22–29)	**< 0.0001**^a^
FVC (% predicted)	87 (71–102)	108 (93–122)	95 (82–105)	72 (69–85)	67 (50–73)	**< 0.0001**^a^
FEV_1_/FVC (%)	52 (41–62)	70 (63–79)	53 (45–60)	43 (39–49)	32 (30–36)	**< 0.0001**^a^
TLC (%predicted)	116 (107–134)	108 (97–126)	114 (108–130)	122 (110–138)	135 (125–139)	0.1027^a^
RV (%predicted)	160 (132–198)	117 (101–147)	153 (135–182)	193 (177–224)	240 (208–268)	**< 0.0001**^a^
RV/TLC (%)	51 (45–57)	45 (38–49)	50 (44–52)	56 (50–63)	68 (56–72)	**< 0.0001**^a^
IC (%predicted)	74 (61–86)	89 (77–110)	79 (74–87)	66 (55–72)	45 (39–57)	**< 0.0001**^a^
FRC (%predicted)	142 (119–165)	109 (99–133)	129 (118–151)	154 (146–188)	193 (166–219)	**< 0.0001**^a^
PREFUL-FV (ml/ml)	0.15 (0.12–0.19)	0.19 (0.14–0.22)	0.16 (0.13–0.19)	0.14 (0.10–0.16)	0.07 (0.07–0.12)	**0.0013**^a^
PREFUL-QCD	0.32 (0.25–0.38)	0.23 (0.21–0.29)	0.31 (0.21–0.34)	0.35 (0.32–0.47)	0.40 (0.38–0.48)	**< 0.0001**^a^
PREFUL-VDP (%)	43 (33–48)	33 (25–39)	41 (30–46)	44 (43–53)	53 (52–57)	**< 0.0001**^a^
PRM-fSAD (%)	33 (18–44)	19 (6–32)	33 (13–40)	38 (29–46)	55 (40–58)	**0.0167**^a^
PRM-emph (%)	4 (0–18)	0 (0–2)	0 (0–12)	11 (2–23)	32 (20–47)	**< 0.0001**^a^
PRM-VDP (%)	42 (23–74)	22 (6–36)	36 (13–53)	53 (42–75)	91 (78 –91)	**< 0.0001**^a^

Unless otherwise indicated, data are median with 25th and 75th percentiles in parentheses; significant differences in bold

*FEV*_1_ forced expiratory volume in 1 s, *FVC* forced vital capacity, *GOLD* global initiative for chronic obstructive lung disease, *TLC* total lung capacity, *RV* reserve volume, *IC* inspiratory capacity, *FRC* functional residual capacity, *A1AD* alpha 1 antitrypsin deficiency, *PREFUL* phase-resolved functional lung, *FV* fractional ventilation, *QCD* quartile coefficient of dispersion, *VDP* ventilation defect percentage, *PRM* parametric response mapping, *fSAD* functional small airway disease, *emph* emphysema

^a^ Kruskal–Wallis test

^b^ Fisher’s exact test

^c^ Exact Kruskal–Wallis test for ordered categorical data

### Validation of down-sampling and section matching

Comparing full resolution vs down-sampled datasets showed a significant, but small bias PRM^normal^ with a mean difference of 0.6% (95% CI: 0.1–1.0%), *p* = 0.004. Significant differences regarding PRM^fSAD^ and PRM^emph^ were not observed. Comparison of PRM parameters of the whole lung compared to the MR-section-matched volume revealed no significant bias (e.g., mean difference for PRM^normal^ 0.8% (95% CI: −1.9–3.4%). *p* = 0.67).

### Global comparison of PREFUL-MRI with PRM

**Fig. 1:**
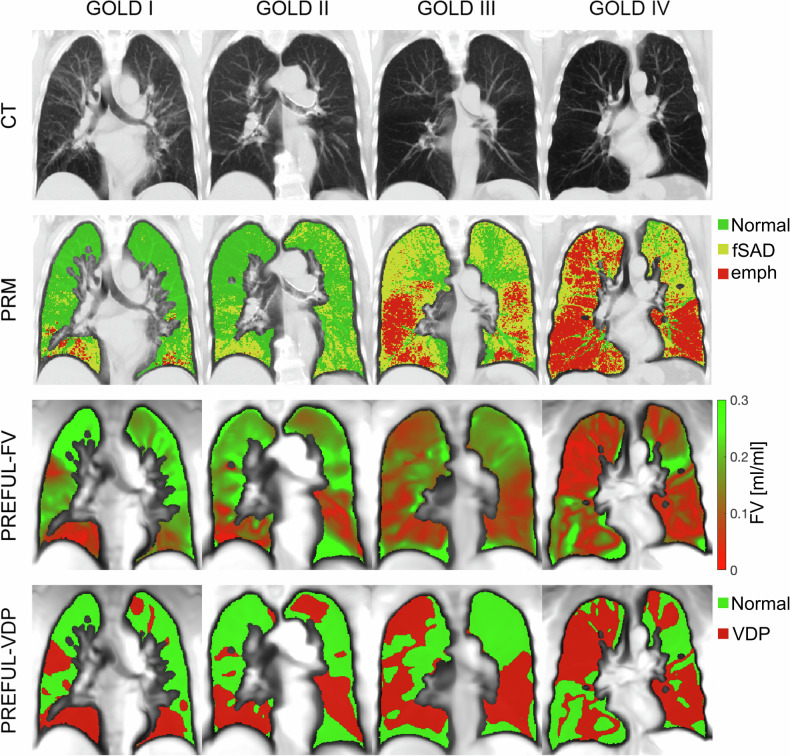
Representative corresponding coronal maps of PRM derived by CT and ventilation defect maps (PREFUL-VDP) calculated from fractional ventilation maps (PREFUL-FV) derived by PREFUL MRI. First column: a 60-year-old man with GOLD I disease (FEV_1_ 91% of predicted value, PRM^normal^ 78%, PRM^fSAD^ 19%, PRM^emph^ 4%, median PREFUL-FV 0.20, PREFUL-VDP 38%, spatial overlap 69%). Second column: a 70-year-old woman with GOLD II disease (FEV_1_ 56% of predicted value, PRM^normal^ 75%, PRM^fSAD^ 25%, PRM^emph^ 0%, median PREFUL-FV 0.20, PREFUL-VDP 39%, spatial overlap 64%). Third column: a 63-year-old woman with GOLD III disease (FEV_1_ 46% of predicted value, PRM^normal^ 25%, PRM^fSAD^ 58%, PRM^emph^ 18%, median PREFUL-FV 0.13, PREFUL-VDP 46%, spatial overlap 56%). Fourth column: a 66-year-old man with GOLD IV disease (FEV^1^ 23% of predicted value, PRM^normal^ 9%, PRM^fSAD^ 40%, PRM^emph^ 52%, median PREFUL-FV 0.07, PREFUL-VDP 58%, spatial overlap 56%)

**Fig. 2:**
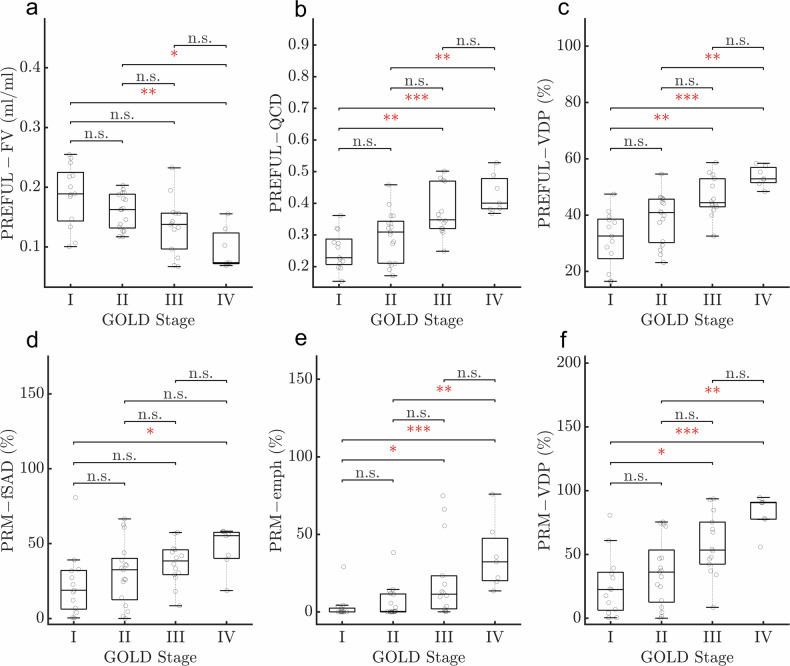
Boxplot representation of PREFUL (top row) and CT (bottom row) parameters across GOLD stages I (*n* = 13), II (*n* = 17), III (*n* = 14), and IV (*n* = 7): PREFUL-FV (**a**), PREFUL-QCD (**b**), PREFUL-VDP (**c**), PRM-fSAD (**d**), PRM-emph (**e**), and PRM-VDP (**f**). Kruskal–Wallis omnibus test was significant for all parameters. Statistical differences between GOLD stages are marked by *p* < 0.05 (*), *p* < 0.01 (**), *p* < 0.001 (***), or non-significant *(*n.s.) results of the Dunn’s test. In each boxplot, the central line denotes the median, the box edges represent the 25th and 75th percentiles, and the whiskers extend to the most extreme values not classified as outliers. Individual data points are shown as circles

**Fig. 3:**
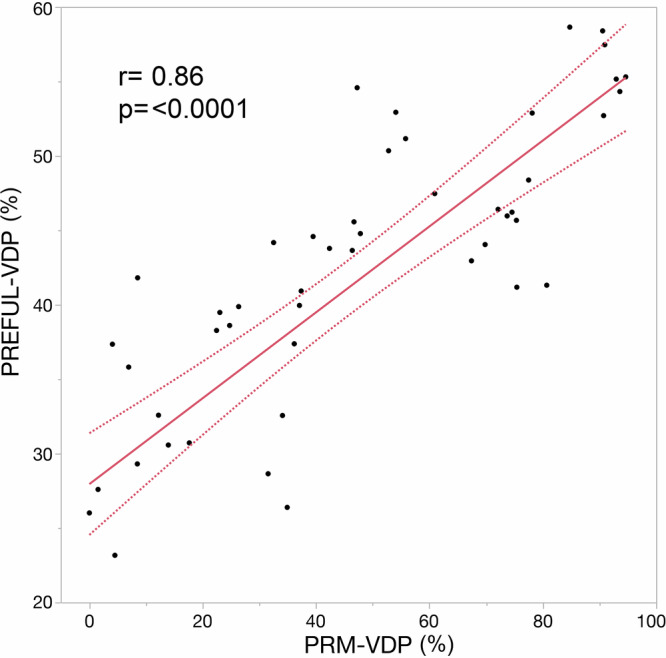
Linear regression comparing VDP assessed with PREFUL and PRM. Intercept: 24%, slope 0.36%, correlation 0.80

Figure 1 shows representative coronal maps of four COPD patients with GOLD stage I–IV with a good spatial agreement of PRM and PREFUL MRI, and demonstrates a visible decrease in lung density with increasing GOLD stage. The quantitative analysis confirmed these observations (Fig. 2): PRM demonstrated increasing fSAD and emphysema (*p* = 0.02 and *p* < 0.001, respectively). In parallel, PREFUL MRI revealed decreasing FV (*p* < 0.01), greater spatial heterogeneity of ventilation QCD (*p* < 0.001), and elevated ventilation defect percentage (PREFUL-VDP; *p* < 0.001) with advancing GOLD stage. As illustrated by Table 2, a moderate correlation between PRM^normal^ and PREFUL-FV was found on a global level (*r* = 0.54, *p* < 0.001), as well as on a lobar level. Stronger correlations were present comparing PRM^normal^ to ventilation heterogeneity (PREFUL-QCD, *r* = −0.72, *p* < 0.001). Correlations of PREFUL-FV and PREFUL-QCD with PRM^fSAD^ and PRM^emph^ were weaker, and in the case of PREFUL-FV and PRM^fSAD^, not significant (e.g., for the whole lung *r* = −0.21 *p* = 1.12). PREFUL-VDP correlated strongly with PRM^fSAD+emph^ (*r* = 0.86, *p* < 0.001), as well as with PRM^fSAD^ and PRM^emph^ separately (Table 3). Linear regression of VDP assessed with PREFUL (*y*-axis) and PRM (*x*-axis) resulted in: Intercept: 25%, slope 0.36%, correlation 0.86, *p* < 0.001 (Fig. 3).

**Table 2:** Comparison of PRM classifications (PRM^Normal^ = 1-PRM-VDP, PRM^fSAD^, PRM^emph^) with PREFUL-derived FV and corresponding QCD as a measure of ventilation heterogeneity

	PREFUL			PRM^normal^			PRM^fSAD^			PRM^emph^		
				%	*r*	*p*	%	*r*	*p*	%	*r*	*p*
Whole lung	FV	0.15 (0.12–0.19)		58 (25–78)	0.54	**< 0.0001**	33 (18–44)	−0.21	0.13	4 (0–18)	−0.47	**0.0006**
	QCD	0.32 (0.25–0.38)			−0.72	**< 0.0001**		0.46	**0.0006**		0.69	**< 0.0001**
Upper lobes	FV	0.16 (0.12–0.20)		62 (29–90)	0.59	**< 0.0001**	27 (10–50)	−0.31	0.03	1 (0–16)	−0.48	**0.0003**
	QCD	0.26 (0.19–0.34)			−0.80	**< 0.0001**		0.56	**< 0.0001**		0.71	**< 0.0001**
Lower lobes	FV	0.14 (0.11–0.17)		46 (23–85)	0.56	**< 0.0001**	38 (15–54)	−0.32	0.02	3 (0–23)	−0.47	**0.0005**
	QCD	0.36 (0.26–0.42)			−0.66	**< 0.0001**		0.48	**0.0004**		0.66	**< 0.0001**
Right lung	FV	0.16 (0.12–0.19)		56 (23–78)	0.58	**< 0.0001**	33 (15–49)	−0.15	0.29	4 (0–20)	−0.55	**< 0.0001**
	QCD	0.30 (0.23–0.36)			−0.74	**< 0.0001**		0.42	**0.003**		0.78	**< 0.0001**
Left lung	FV	0.15 (0.11–0.18)		61 (30–81)	0.50	**0.0002**	33 (19–50)	−0.30	0.03	2 (0–16)	−0.31	0.03
	QCD	0.32 (0.22–0.39)			−0.72	**< 0.0001**		0.57	**< 0.0001**		0.59	**< 0.0001**
Right upper lobe	FV	0.16 (0.12–0.21)		59 (24–92)	0.60	**< 0.0001**	29 (8–51)	−0.27	0.06	1 (0–21)	−0.54	**< 0.0001**
	QCD	0.24 (0.17–0.32)			−0.80	**< 0.0001**		0.52	**< 0.0001**		0.77	**< 0.0001**
Right lower lobe	FV	0.14 (0.11–0.17)		46 (21–82)	0.56	**< 0.0001**	39 (16–49)	−0.30	0.03	2 (0–27)	−0.49	**0.0003**
	QCD	0.34 (0.25–0.43)			−0.66	**< 0.0001**		0.34	0.01		0.70	**< 0.0001**
Left upper lobe	FV	0.16 (0.11–0.20)		66 (33–90)	0.43	**0.002**	23 (10–46)	−0.24	0.08	1 (0–9)	−0.31	0.03
	QCD	0.25 (0.20–0.32)			−0.73	**< 0.0001**		0.55	**< 0.0001**		0.65	**< 0.0001**
Left lower lobe	FV	0.13 (0.10–0.17)		51 (25–82)	0.46	**0.0008**	36 (18–56)	−0.26	0.06	3 (0–24)	−0.29	0.04
	QCD	0.34 (0.25–0.45)			−0.63	**< 0.0001**		0.57	**< 0.0001**		0.55	**< 0.0001**

Unless otherwise indicated, data are median with 25th and 75th percentiles in parentheses; significant *p*-values after Bonferroni correction are indicated in bold (*p* < 0.006)

*fSAD* functional small airways disease, *emph* emphysema, *FV* fractional ventilation, *QCD* quartile coefficient of dispersion, *PREFUL* phase-resolved functional lung, *PRM* parametric response mapping

**Table 3:** Comparison of VDP derived from PREFUL-FV and PRM measures

PREFUL	PRM		Spearman’s correlation			Spatial overlap		
VDP (%)	parameter	VDP (%)	*r*	descriptive *p*	bonferroni *p*	Overlap (%)	Dice coefficient (healthy)	Dice coefficient (defect)
42.9 (32.6–48.4)	fSAD + emh	42 (23–75)	0.86	< 0.0001	< 0.0001	60 (55–66)	0.69 (0.39–0.74)	0.53 (0.28–0.62)
	fSAD	33 (18–44)	0.57	< 0.0001	0.0001	60 (51–65)	0.69 (0.51–0.74)	0.42 (0.27–0.54)
	emph	4 (0.1–18)	0.82	< 0.0001	< 0.0001	58 (55–67)	0.72 (0.65–0.81)	0.01 (0.00–0.31)

Unless otherwise indicated, data are median with 25th and 75th percentiles in parenthesis

*fSAD* functional small airways disease, *emph* emphysema, *VDP* ventilation defect percentage, *QCD* quartile coefficient of dispersion, *PREFUL* phase-resolved functional lung, *PRM* parametric response mapping

### Regional comparison of PREFUL-MRI and PRM

Spatial overlap (i.e., percentage of voxels labeled as ventilation defect or healthy lung parenchyma with both methods) showed an agreement of 60% for the whole lung with a corresponding dice coefficient of 53% (28–62%) for the defect voxels and 68% (39–74%) for normal ventilation voxels.

**Fig. 4:**
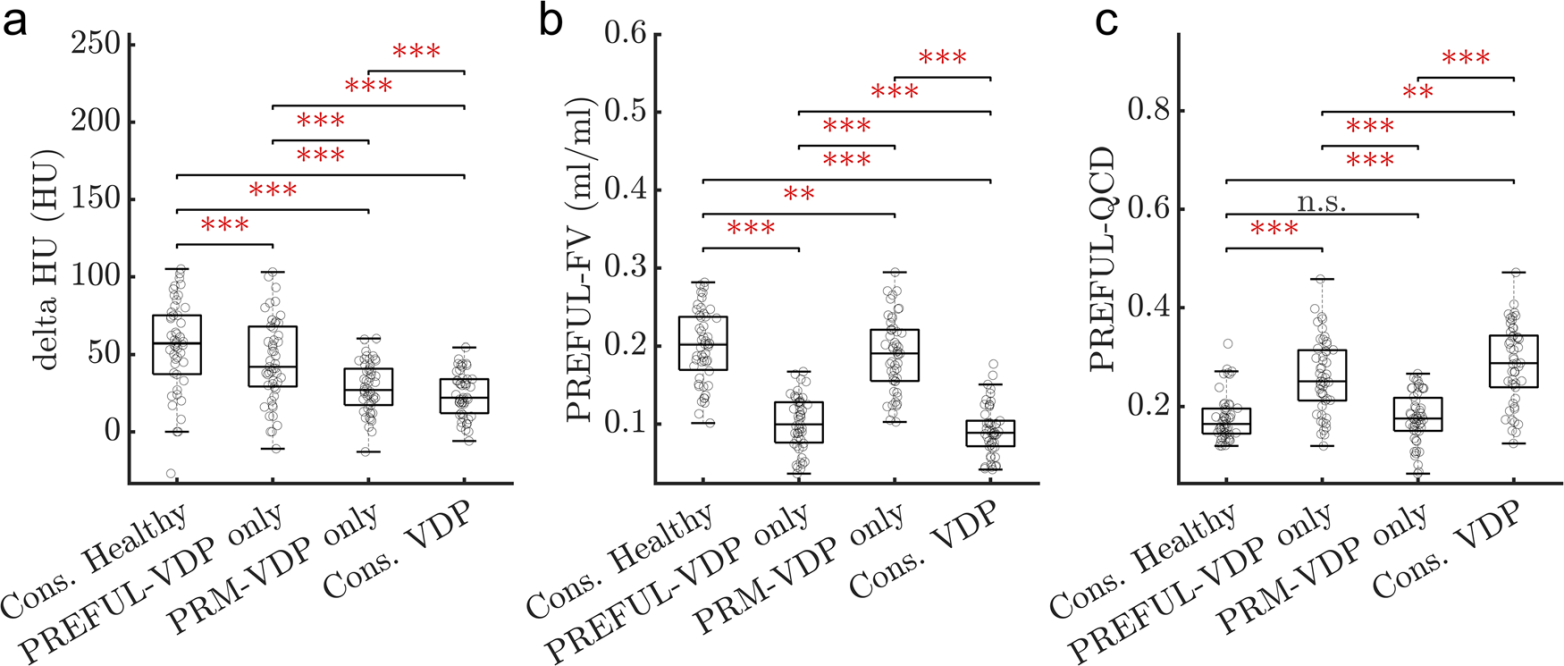
Comparison of spatial con- and discordance between PREFUL and PRM and their influence on the corresponding distribution of ΔHounsfield units (HU) between expiration and inspiration (**a**), PREFUL-FV (**b**), and PREFUL-QCD (**c**). Cons. healthy, consensus healthy; Cons. VDP, consensus VDP. Statistical differences are marked by *p* < 0.05 (*), *p* < 0.01 (**), *p* < 0.001 (***), or non-significant (n.s.)

**Fig. 5:**
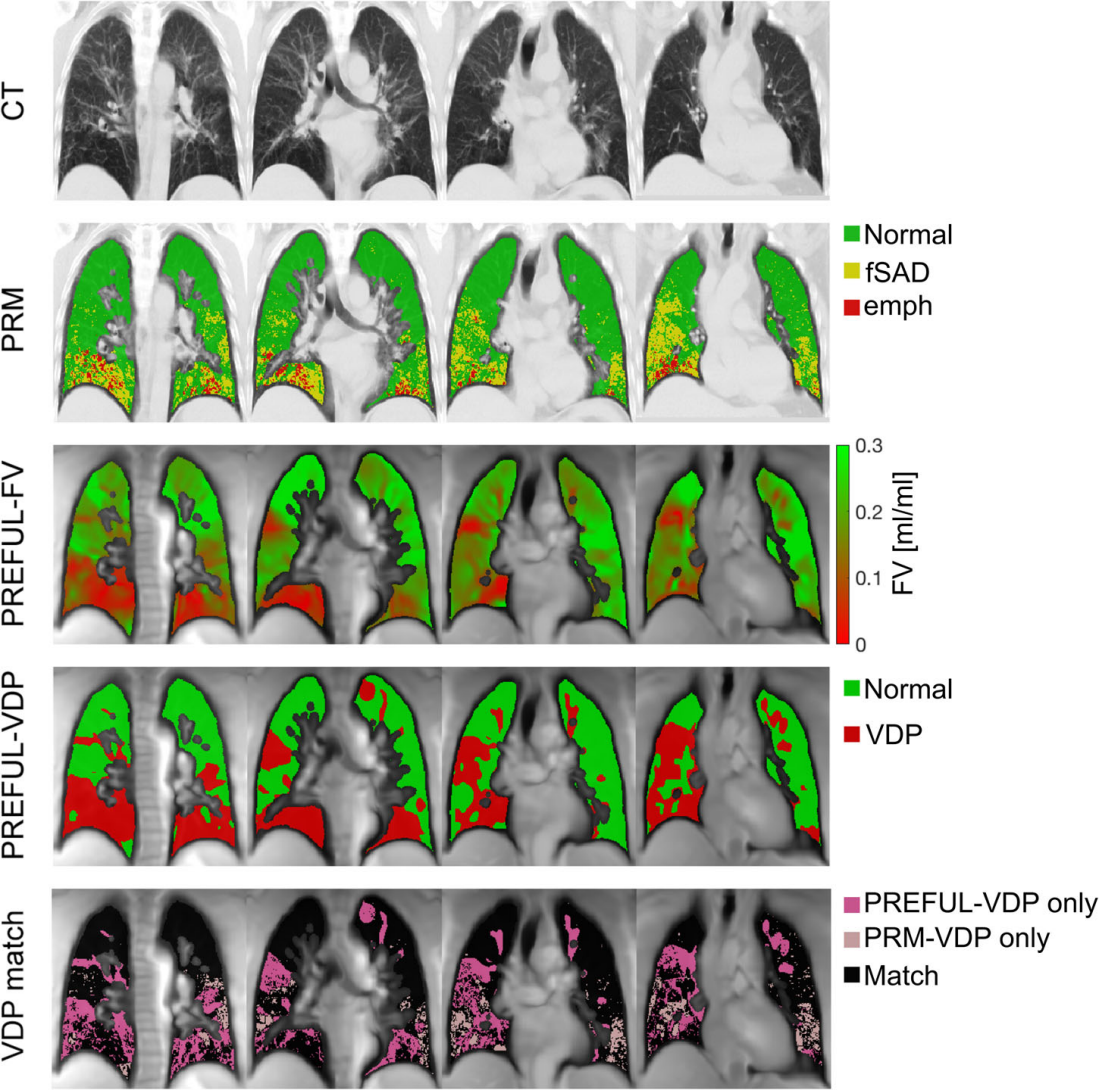
Representative coronal maps of a 60-year-old man with GOLD I disease (FEV_1_ 91% of predicted value, PRM^normal^ 78%, PRM^fSAD^ 19%, PRM^emph^ 4%, PRM^fSAD+emph^ 23%, median PREFUL-FV 0.20, PREFUL-VDP 38%, spatial overlap 69%). First row: CT; second row: parametric response maps (PRM); third row: FV derived by PREFUL MRI; fourth row: ventilation defect map derived by PREFUL MRI; fifth row: black: matching results of both methods, pink: ventilation defect detected by PRM only, purple: ventilation defect detected by PREFUL MRI only

PREFUL-FV and QCD of PRM-VDP only-areas were significantly lower compared to consensus healthy-areas and significantly higher than consensus VDP-areas (*p* < 0.001). Voxels labeled as PREFUL-VDP only showed significantly reduced ΔHU compared to consensus healthy-areas but significantly higher values than consensus VDP-areas (*p* < 0.001). Analogous to that, these findings were also true for PREFUL-FV and -QCD. See Fig. 4 for a boxplot and Table 4 for a numeric summary of consensus-analysis and Fig. 5 for a representative case.

**Table 4:** Comparison of PREFUL and CT parameters in regions with con- and discordant labeled ventilation defects

		CT			PREFUL	
	% of total	Expiration (HU)	Inspiration (HU)	Delta HU	FV (mL/mL)	QCD
Cons. healthy	39 (16–54)	−808 (−823 to −794)	−872 (−888 to −859)	57 (37-75)	0.20 (0.17-0.24)	0.17 (0.15-0.20)
PREFUL-VDP only	19 (10–25)	−815 (−825 to −802)	−869 (−885 to −857)	42 (29–68)	0.10 (0.08–0.13)	0.25 (0.21–0.32)
PRM-VDP only	20 (9–37)	−883 (−901 to −870)	−918 (−931 to −909)	27 (17–41)	0.19 (0.16–0.22)	0.18 (0.15–0.22)
Cons. VDP	25 (8–36)	−887 (−910 to −873)	−919 (−934 to −909)	22 (12–34)	0.09 (0.07–0.11)	0.29 (0.23–0.35)
Omnibus P	**1.89e–04**	**4.13e–27**	**4.59e–24**	**8.09e–22**	**3.41e–28**	**2.01e–17**
P (Cons. Healthy vs PREFUL–VDP only)	**3.14e–09**	**1.99e–04**	1.76e–01	**1.57e–07**	**5.15e–10**	**1.25e–09**
P (Cons. Healthy vs PRM–VDP only)	**1.05e–02**	**5.14e–10**	**8.62e–09**	**2.08e–09**	**3.45e–03**	9.33e–01
P (Cons. Healthy vs Cons. VDP)	**3.34e–02**	**9.64e–09**	**1.12e–07**	**2.34e–09**	**5.15e–10**	**7.32e–09**
P (PREFUL–VDP only vs PRM–VDP only)	1.63e–01	**5.12e–10**	**3.31e–09**	**1.75e–09**	**5.15e–10**	**2.87e–08**
P(PREFUL–VDP only vs Cons. VDP)	1.20e–01	**9.62e–09**	**2.79e–08**	**1.98e–09**	**1.15e–06**	**7.14e–03**
P (PRM–VDP only vs Cons. VDP)	1.20e–01	**3.76e–05**	8.26e–02	**8.55e–06**	**5.15e–10**	**8.18e–09**

Unless otherwise indicated, data are median with 25th and 75th percentiles in parenthesis; Significant *p*-values were indicated bold (*p* < 0.05)

*VDP* ventilation defect percentage, *HU* Hounsfield units, *delta HU* HU difference between expiration and inspiration, *FV* fractional ventilation, *cons.* consensus, *QCD* quartile coefficient of dispersion, *PRM* parametric response mapping, *PREFUL* phase-resolved functional lung, *VDP* ventilation defect percentage

### Correlation with spirometry

Linear regressions for PRM and the PREFUL parameters showed significant correlations with lung function parameters (Table 5). Strong significant correlations were found between PREFUL-VDP and FEV_1_ (% predicted), *r* = 0.75, *p* < 0.001, and FEV_1_/forced vital capacity (FVC). *R* = 0.73, *p* < 0.001 and RV (% predicted) *r* = 0.64, *p* < 0.001.

**Table 5:** Correlation of PRM and PREFUL with lung function parameters

		Lung function test							
		FEV_1_ (% predicted)	FVC (% predicted)	FEV_1_/FVC (%)	TLC (% predicted)	RV (% predicted)	RV/TLC (%)	IC (% predicted)	FRC (% predicted)
CT									
PRM^normal^	*r*	0.69	0.36	0.72	−0.53	−0.64	−0.56	0.62	−0.73
	desc. *p*	**< 0.0001**	0.009	**< 0.0001**	**< 0.0001**	**< 0.0001**	**< 0.0001**	**< 0.0001**	**< 0.0001**
PRM^fSAD^	*r*	−0.48	−0.31	−0.47	0.21	0.35	0.32	−0.43	0.40
	desc. *p*	**0.0003**	0.03	**0.0005**	0.14	0.01	0.02	**0.002**	**0.003**
PRM^emph^	*r*	−0.70	−0.33	−0.76	0.52	0.61	0.42	−0.58	0.71
	desc. *p*	**< 0.0001**	**0.002**	**< 0.0001**	**< 0.0001**	**< 0.0001**	**0.002**	**< 0.0001**	**< 0.0001**
PREFUL									
FV	*r*	0.60	0.31	0.59	−0.58	−0.72	−0.69	0.43	−0.71
	desc. *p*	**< 0.0001**	0.03	**< 0.0001**	**< 0.0001**	**< 0.0001**	**< 0.0001**	0.002	**< 0.0001**
QCD	*r*	−0.72	−0.53	−0.65	0.44	0.65	0.61	−0.62	0.67
	desc. *p*	**< 0.0001**	**< 0.0001**	**< 0.0001**	**0.001**	**< 0.0001**	**< 0.0001**	**< 0.0001**	**< 0.0001**
VDP	*r*	−0.75	−0.48	−0.73	0.45	0.64	0.57	−0.63	0.70
	desc. *p*	**< 0.0001**	**0.0003**	**< 0.0001**	**0.0009**	**< 0.0001**	**< 0.0001**	**< 0.0001**	**< 0.0001**

Parameters were compared by using the Spearman correlation coefficient. Significant *p*-values after Bonferroni correction (*n* = 9) were indicated in bold (*p* < 0.006)

*QCD* quartile coefficient of dispersion, *QDP* perfusion defect percentage, *FEV*_1_ forced expiratory volume in 1 s, *FVC* forced vital capacity, *TLC* total lung capacity, *RV* reserve volume, *IC* inspiratory capacity, *FRC* functional residual capacity, *fSAD* functional small airways disease, *emph* emphysema, *VDP* ventilation defect percentage, *FV* fractional ventilation, *QCD* quartile coefficient of dispersion, *PREFUL* phase-resolved functional lung, *PRM* parametric response mapping

## Discussion

Data were analyzed from a prospective, single-center investigation embedded in the COSYCONET cohort. The retrospective analysis focused on the agreement between PREFUL MRI and CT–based PRM in 51 clinically stable individuals with COPD. The main results are: Regional assessment of ventilation defects, as well as ventilation heterogeneity using PREFUL MRI, significantly correlated best with the volume of fSAD and emphysema combined derived by PRM CT in COPD patients. A decrease of PREFUL-FV and an increase of PREFUL-VDP were observed with increasing GOLD stage. Both methods correlated well with lung function test parameters such as FEV_1_, FEV_1_/FVC, and RV in COPD patients.

PRM was developed to improve the measurement of fSAD and emphysema by the spatial combination of two thresholds, providing a robust biomarker for COPD phenotyping. Image coregistration is used to classify each voxel by means of its radiodensity in inspiration and expiration, allowing to distinguish the contributions of small airways disease and emphysema from the gas-trapping measurements. Furthermore, spatial agreement with MRI-assessed perfusion analysis was demonstrated recently [37].

PREFUL-derived FV, VDP, as well as QCD as a measurement of ventilation heterogeneity, correlated most strongly with the combination of PRM^fSAD^ and PRM^emph^ rather than with either component alone. This is plausible because FV and its derived metrics are inherently relative ventilation measures: while they likely reflect absolute parenchymal density to some degree, they cannot directly differentiate between fSAD and emphysema, as PRM can. Notably, Capaldi et al compared ^3^He MR imaging-derived VDP with PRM in COPD patients, also reporting a weaker correlation of VDP with PRM^fSAD^ (Person’s *r* = 0.47, *p* = 0.03) compared to PRM^emph^ (Person’s *r *= 0.62, *p* < 0.001) [13].

The calculation of PREFUL-VDP resulted in an even stronger agreement of PREFUL and PRM compared to PREFUL-FV. This was not unexpected since the calculation of binary defect maps reduces the local variation of values. In a recent study, semiautomatically calculated VDPs assessed by FD MRI were compared with a CT-based quantification of emphysema below −950 HU (the same threshold as used for PRM^emph^) and VDP derived from pulmonary hyperpolarized ^3^He MRI in 12 COPD patients and reported that FD-VDP strongly correlated globally and spatially both with CT (Pearson *r* = 0.80, *p* = 0.002) and ^3^He-MRI VDP [24] (*r* = 0.88; *p* < 0.001). This is in line with the current study showing a significant correlation of PREFUL-VDP and PRM^emph^ with a Spearman *r* = 0.82, *p* < 0.001. Furthermore, very similar results were reported by Friedlander et al in severe asthma [29], where PREFUL-VDP correlated with PRM^fSAD^ (*r* = 0.55), closely matching our observed correlation (*r* = 0.57).

Despite the strong correlations, further comparison of PREFUL and PRM revealed systematic differences between the two techniques. First, the regression intercept showed that PREFUL-VDP was already at ~25% even when PRM^fSAD+emph^ was 0%. Second, the low regression slope suggests that PREFUL may not capture the full extent of disease in severe COPD. These discrepancies are likely related to the adaptive thresholding approach used to calculate PREFUL-VDP. In our study, the 75th percentile of the signal distribution, assumed to represent healthy lung tissue, was used as a reference and scaled by an empirical factor of 0.7. This empirical factor appears to overestimate disease burden in early COPD, while reliance on the 75th percentile underestimates disease burden in advanced COPD, where only limited healthy reference tissue remains. Further optimization of the thresholding strategy is therefore warranted.

Additional factors may also contribute to differences between PRM and PREFUL: (1) the relatively large slice thickness and lower in-plane resolution of PREFUL MRI compared with CT likely reduce sensitivity for detecting small ventilation defects, reflecting the trade-off between temporal resolution and signal-to-noise ratio.

(2) PREFUL-MRI is performed during tidal breathing, whereas CT-based PRM uses scans at RV and TLC. Forced respiratory maneuvers can accentuate disease, similar to how forced expiration is required to measure FEV₁. This is supported by Comellas et al, who demonstrated that the amount of fSAD changed substantially when PRM was applied at functional residual capacity rather than RV, likely due to incomplete emptying of air in the voxels. Moreover, PRM at RV provided stronger differentiation between participants with and without COPD [38]. Whether the sensitivity of PREFUL can be enhanced by including forced respiratory maneuvers remains to be determined; preliminary data suggest potential for this approach [39], although in this case, the major advantage of PREFUL - its independence from patient cooperation - would be diminished.

Beyond the quantitative and correlation comparison, the spatial agreement between PREFUL MRI and PRM was also analyzed. For the defect class, the obtained Dice coefficient of 0.53 corresponds to an overlap of approximately 23% of the lung voxels between both modalities, given the observed defect fractions in this cohort. This level of overlap is substantially above chance, which would be expected to be markedly lower for the same defect fractions and ROI size. A detailed quantitative derivation and statistical interpretation are provided in the supplementary material.

Concordant and discordant ventilation defects identified by PREFUL MRI and PRM were further analyzed using match-/mismatch-maps. Notably, voxels classified as defective by PREFUL MRI but not by PRM exhibited significantly different ΔHU values compared with regions labeled as healthy or defective by both modalities. Conversely, voxels identified as defective only by PRM showed PREFUL-FV and QCD values that were significantly lower than in healthy regions but higher than in areas classified as defective by both methods. Taken together, these findings highlight the limitations of binary classifications: although regions were not consistently aligned between methods, they demonstrated similar trends, suggesting that threshold choice strongly influences classification, sensitivity, and ultimately the degree of concordance. In this context, the introduction of continuous probability maps (e.g., probability values for fSAD rather than binary labels), as proposed by Kirby et al, may provide a more robust alternative by capturing intermediate states and reducing threshold dependency [40].

### Limitations

This study has several limitations. First, CT imaging with reduced dose settings (equivalent dose of < 3.5 mSv), as applied here, is known to bias PRM measurements toward higher emphysema and fSAD percentages, though the magnitude of this effect remains uncertain [41]. Nevertheless, the PRM values obtained in our study were consistent with previously published data where comparisons were possible [41]. Second, CT scans were not spirometrically controlled. Therefore, since varying respiratory effort can affect the reproducibility of image registration metrics, participants were carefully coached to achieve maximal inspiration and end expiration.

Third, image artifacts—particularly from cardiac motion in the lower lobes—and potential errors in intermodal registration between CT and PREFUL may have reduced the observed correlation between both techniques. Despite this, significant associations between PREFUL MRI and PRM were demonstrated.

Fourth, although the COSYCONET cohort study is a multicenter, longitudinal project including a large number of patients across Germany, the imaging substudy analyzed here was performed at a single center and included only one imaging time point so far. PREFUL MRI was introduced as a local protocol amendment rather than as a standardized multicenter approach.

Finally, the median time interval between MRI and lung function testing was approximately one month, which may have introduced bias into the correlation analysis. In future studies, pulmonary function testing should ideally be performed on the same day as MRI. Nevertheless, significant correlations between PREFUL MRI, PRM, and spirometry were observed in this cohort of stable COPD patients.

## Conclusion

PREFUL-VDP showed the strongest correlation with PRM measures of emphysema and functional small airways disease combined. These findings support PREFUL as a promising, noninvasive, radiation-free tool for assessing and monitoring regional ventilation in patients with COPD.

## Supplementary information

ELECTRONIC SUPPLEMENTARY MATERIAL

## Abbreviations

^3^He: Helium-3
^19^F: Fluorine-19
^129^Xe: Xenon-129
ANTs: Advanced normalization tools
CI: Confidence interval
COPD: Chronic obstructive pulmonary disease
COSYCONET: COPD and SYstemic consequenzes–COmorbidities NETwork
ΔHU: Difference in Hounsfield units between expiration and inspiration
FD: Fourier decomposition
FEV₁: Forced expiratory volume in 1 s
FV: Fractional ventilation
FVC: Forced vital capacity
GOLD: Global initiative for chronic obstructive lung disease
HU: Hounsfield unit
PREFUL: Phase-resolved functional lung
PREFUL-FV: PREFUL fractional ventilation
PREFUL-QCD: PREFUL quartile coefficient of dispersion (ventilation heterogeneity metric)
PREFUL-VDP: PREFUL ventilation defect percentage
PRM: Parametric response mapping
PRM^emph^: PRM emphysema
PRM^fSAD^: PRM functional small airways disease
PRM^normal^: PRM normal lung parenchyma
PRM-VDP: PRM ventilation defect percentage (PRM^fSAD^ + PRM^emph^)
QCD: Quartile coefficient of dispersion
RV: Residual volume
*S*_Exp_: PREFUL MRI signal at end-expiration
*S*_Insp_: PREFUL MRI signal at end-inspiration
TLC: Total lung capacity
VDP: Ventilation defect percentage
