# Supplementary material for: Comparison of phase-resolved functional lung (PREFUL) MRI and CT parametric response mapping (PRM) in COSYCONET COPD
Source: Eur Radiol. 2026 Mar 28;36(7):5454–68. doi: 10.1007/s00330-026-12429-3 (PMC13282224; doi:10.1007/s00330-026-12429-3)
Supplement: Supplementary file 1 — ELECTRONIC SUPPLEMENTARY MATERIAL [file 330_2026_12429_MOESM1_ESM.pdf]

# Comparison of phase-resolved functional lung (PREFUL) MRI and CT parametric response mapping (PRM) in COSYCONET COPD

# ELECTRONIC SUPPLEMENTARY MATERIAL

## Supplementary Appendix A — Interpretation of Dice Coefficient Agreement

Direct interpretation of Dice values is challenging, especially for defect classes, as defect regions occupy typically substantially smaller volumes than normal lung, and small spatial misalignments disproportionately affect the Dice score. Consequently, moderate absolute Dice values are typical even when biological agreement is good.

To address whether the observed Dice coefficient for the defect class is "close to chance," a quantitative interpretation based on the algebraic relationship between Dice and voxel overlap and a probability assessment using the hypergeometric distribution is provided:

The Dice coefficient is defined as

$$\text{Dice} = 2|X \cap Y| / (|X| + |Y|),$$

where X and Y denote the defect regions from PREFUL-VDP and PRM-VDP. A defect DICE of 0.53 therefore means that the overlap between both defect maps corresponds to 53% of the average defect volume. Relating this to the whole lung ROI,

$$|X \cap Y| / N = (\text{Dice} / 2)(p + q),$$

with p = 0.43 (PREFUL-VDP) and q = 0.42 (PRM-VDP), yields an overlap of 23% of all lung voxels (= 3,450 voxels for a representative slice with N = 15,000 lung voxels).

The null hypothesis of random PREFUL-VDP placement corresponds to sampling without replacement and is described by a hypergeometric distribution.

With |X| = 6,450, |Y| = 6,300 and N = 15,000, and K_obs = 3,450, the tail probability for:

$$P(K \geq K\_obs) = 1 - P(K \leq K\_obs - 1),$$

computed via MATLAB's hygecdf is on the order of $10^{-111}$, i.e. effectively zero. Thus, while a Dice of 0.53 may appear moderate in absolute terms, it is far above what would be expected by chance for the observed defect fractions and ROI size.

This is further confirmed by the hypergeometric expectation,

$$E[K] = |X| \cdot |Y| / N = 6450 \cdot 6300 / 15000 = 2709,$$

which is far below the observed value.
